# Anaphylaxis

**Published:** 1911-02-11

**Authors:** 


### The Hospital

A Journal of

The Medical Sciences and Hospital Administration.

New Series. No. 210, Vol. VIII. [No. 1282, Vol XLIX.] SATURDAY, FEBRUARY 11, 1911.

### Anaphylaxis.

We have been accustomed for many years, as the result of clinical experience, to expect that one attack of a specific disease, such, for instance, as measles or scarlet fever, will act as a preventive to a second attack in the same subject, the development of antibodies in the particular patient's serum having produced an active immunity. In the case of small-pox, indeed, so well was this known two centuries ago that inoculation of the virus was at one time largely employed, so that the patient might get the disease over when healthy, and at a time convenient to himself; and vaccination, which drove this older method out of use, is now known to be only another way of introducing an attenuated virus of the same disease, capable of producing a similarly active though not so persistent an immunity. As a corollary to the production of this active immunity it has been found that the serum of such an immune animal, when injected into one not immune, can confer, in some instances, on that animal immunity also, this passive immunity, so acquired, being the underlying principle of the serum treatment of diphtheria, etc. So accustomed have our minds become to these phenomena that it comes almost as a shock to find there is a reverse side to the picture, that in some individuals injection of certain substances, instead of producing toleration, makes them hypersensitive to a second dose, so that even a tiny repeated injection, innocuous to a normal individual, can produce in these sensitised people distressingly grave and even fatal symptoms.

This condition of hypersensitisation is known as "Anaphylaxis," and it may probably help to explain some of the mysteries associated with the words "idiosyncrasy" and "idiopathic." According to Armand Deville, "there exists a certain number of substances which, introduced for the first time into an organism in innocuous doses, have the property of producing in that organism, after a fixed incubation period, an extreme sensitiveness to minute doses of the same substance, which would produce no effect whatever in another organism not so sensitised." Among such substances are extract of shell-fish, actinia poison, certain foreign proteins such as anti-diphtheritic serum, the pollen of plants, and various drugs such as antipyrin and potassium iodide.

Amongst the earlier experiments on anaphylaxis were those of Richet and Theobald Smith. Richet, working with actinia poison, found that a second smaller injection in an animal was much more toxic than the first, and Theobald Smith found experimentally that if a normal guinea-pig was injected with 5 c.c. of horse serum no result followed, but if the same animal had had a minute injection of the same serum fourteen days before it in most cases succumbed. Since these preliminary experiments much work has been done, and it is now generally conceded that previous susceptibility is necessary for the production of anaphylaxis. This susceptibility may be experimentally obtained in animals by the injection of foreign protein: albumen, serum, actinia extract.

The symptoms vary from mild local effects to intense constitutional symptoms, sometimes ending in death. Locally there is redness, swelling, and wheals, at the seat of injection (Arthus phenomenon). Constitutionally, within a few minutes in guinea-pigs, there is restlessness, coughing, sneezing, apparent swelling of the mucous membrane of the nose and pharynx, irregular rapid respirations and dyspnœa. This is followed by paresis, and if at this stage the animal does not recover, respiratory paralysis, convulsions, and death ensue. A similar sequence of events has occurred in the human subject as the result of a second injection of antidiphtheritic serum some days after the first. The condition was described by Von Pirquet and Schick under the name of "Serum disease," and is a typical example of anaphylaxis. Most of the cases are mild, but in a well-marked condition the symptoms are those of some skin eruption, exudative or urticarial, transitory œdema, often of the angioneurotic variety, extreme itchiness, swelling of the mucous membrane of the upper respiratory passages, albuminuria, painful swelling of joints, and in violent cases dyspnœa, cyanosis, convulsions, paralysis, and death. Gillete, in the *New York State Journal of Medicine* for September 1909, collected and reported thirty cases, with sixteen deaths in from five to fifteen minutes after injection, the symptoms in all the fatal cases being alarming dyspnœa, urticaria, and collapse. Some of the patients were asthmatics, and it is worthy of note that Billard (*Lancet*, October 22, 1910) classes urticaria, hay fever, and spasmodic asthma, as anaphylactic diseases produced by the presence of minute quantities of foreign protein in an organism already sensitised by previous contact. It is probable that "serum fever" is more common than is supposed, and that a good many of the cases recorded as scarlet fever following diphtheria are in reality unrecognised cases of a mild type, desquamation after the urticarial eruption being taken for the more common disease. As to the mechanism by which anaphylaxis is brought about very little is known at present. Moschcowitz in a recent paper (*New York Medical Journal*, January 7) points out the constant presence of eosinophilia in most of the recognised anaphylactic diseases. What connection, however, these cells have with the phenomena of anaphylaxis, either of a causative or consecutive nature, still remains obscure, and how the condition can be diagnosed and guarded against in apparently normal individuals has not yet been worked out.

It is, however, a distinct advance to know that such a condition does exist, and that the puzzle of "idiosyncrasy" appears somewhat nearer solution by the new light thus let in upon it, which, however, will not please certain extremists.

